# ROS Induced by *Aphrocallistes vastus* Lectin Enhance Oncolytic Vaccinia Virus Replication and Induce Apoptosis in Hepatocellular Carcinoma Cells

**DOI:** 10.3390/md22070307

**Published:** 2024-06-30

**Authors:** Yanan Zhang, Ying Zhu, Gaohui Jiang, Ke Chen, Guohui Zhang, Kan Chen, Ting Ye, Yanrong Zhou, Gongchu Li

**Affiliations:** College of Life Sciences and Medicine, Zhejiang Sci-Tech University, Hangzhou 310018, China

**Keywords:** ROS, *Aphrocallistes vastus* lectin, oncolytic vaccinia virus

## Abstract

Oncolytic virotherapy is expected to provide a new treatment strategy for cancer. *Aphrocallistes vastus* lectin (AVL) is a Ca^2+^-dependent lectin receptor containing the conserved domain of C-type lectin and the hydrophobic N-terminal region, which can bind to the bird’s nest glycoprotein and D-galactose. Our previous studies suggested that the oncolytic vaccinia virus (oncoVV) armed with the AVL gene exerted remarkable replication and antitumor effects in vitro and in vivo. In this study, we found that oncoVV-AVL may reprogram the metabolism of hepatocellular carcinoma cells to promote ROS, and elevated ROS subsequently promoted viral replication and induced apoptosis. This study will provide a new theoretical basis for the application of oncoVV-AVL in liver cancer.

## 1. Introduction

Lectins, ubiquitously distributed in plants, animals, and fungi, are highly diverse carbohydrate-binding proteins that selectively recognize and bind distinct different sugar-containing receptors on cellular surfaces [1]. Many lines of evidence suggest that lectins induce cell damage or death by mediating redox systems. For example, *Galectin-3* lectin caused damage and even death to ARPE-19 cells through oxidative stress [2]. *Pinellia pedatisecta* lectin stimulated macrophages to reduce mitochondrial membrane potential, leading to up-regulation of the ROS (reactive oxygen species)-MAPKs/NF-κB pathway, and inducing pyroptosis of RAW264.7 cells [3]. The lectin-like oxidized low-density lipoprotein receptor LOX-1 induced apoptosis of vascular smooth muscle cells by activating pro-apoptotic factors Caspase-3/Caspase-9 through oxidative stress [4]. Furthermore, *cystosericin* lectin induced autophagic death of A375 melanoma cells through the ROS-p38-p53 pathway [5]. 

*Aphrocallistes vastus* lectin (AVL) is a C-type lectin from hexactinellid sponges with a size of 34 kDa. The process of AVL aggregation is Ca^2+^-dependent, which was inhibited by bird’s nest glycoproteins and D-galactose. It has been shown that AVL is a cell adhesion molecule that may bind to the cell membrane through the hydrophobic portion and interact with carbohydrate structures on the surfaces of other cells or cell vesicles [6,7]. 

Oncolytic virus therapy appears to be the new frontier in cancer treatment, following the success of immunotherapy using immune checkpoint inhibitors [8]. Oncolytic viruses were a type of natural or genetically modified viruses that target and multiply within cancer cells, causing cell destruction while avoiding harm to healthy tissue [9]. Various oncolytic virus vectors, such as herpes simplex virus, adenovirus, Newcastle disease virus, poliovirus, vaccinia virus, reovirus, and parvovirus, have been extensively researched in both preclinical investigations and clinical trials. OncoVV virus is known for its extensive DNA genome, which enables the easier manipulation and packaging of larger transgenes.

Viruses have evolved to hijack cellular anabolic pathways to replicate [10], and increasing evidence suggests that increasing the flow of the TCA cycle creates a state that is conducive to virus replication [11]. In fact, increasing metabolic flux through carboxylation of glutamine to α-ketoglutaric acid(α-KG) enhanced the oncolytic properties of adenovirus [12] and human cytomegalovirus (HCMV) [13]. Etomoxir or trimetazidine diminished poxvirus replication by inhibiting fatty acid β-oxidation and preventing entry into the TCA cycle [14]. In particular, the RIG-I-MDA5-MAVS complex was the primary antiviral defense system bound directly to the mitochondrial membrane [15].

Several viruses have been found to utilize oxidative phosphorylation, in which electrons are transferred from reduced molecules NADH or FADH2 to oxygen inside the mitochondria, resulting in the production of ATP [16]. For example, lysosomal human cytomegalovirus [17] and rubella virus [18] increased oxidative phosphorylation, whereas inhibition or reduction of oxidative phosphorylation inhibited hepatitis B virus replication [19]. Furthermore, the replication of viruses and the lysis of tumors are also influenced by reactive oxygen species (ROS), which are generated as a result of oxidative phosphorylation [20]. ROS enhanced the cytotoxicity induced by avian reovirus echoviruses [21], hepatitis C virus [22], and Epstein–Barr virus [23]. ROS activated high mobility group box 1(HMGB1) and improved respiratory syncytial virus (RSV) lysis [24]. 

The oncoVV utilized in this study is a version of the Western Reserve (WR) strain virus that has been modified to remove the viral thymidine kinase (TK) gene [25]. Our previous studies have found that the oncolytic vaccinia virus expressing *Aphrocallistes vastus* lectin (oncoVV-AVL) exerted remarkable replication and antitumor effects in vitro and in vivo. Furthermore, oncoVV-AVL has shown a good safety profile in animal transplantation tumor models [26]. However, the particular mechanism requires additional investigation. In the presented studies, we demonstrated that the AVL gene can be expressed in hepatocellular carcinoma (HCC) cells (Figure S1). Moreover, we found that oncoVV-AVL can reprogram the metabolism of HCC cells to promote ROS, and elevated ROS subsequently promoted viral replication and induced apoptosis.

## 2. Results

### 2.1. OncoVV-AVL Enhanced ROS Formation in HCC Cells

**Figure 1:**
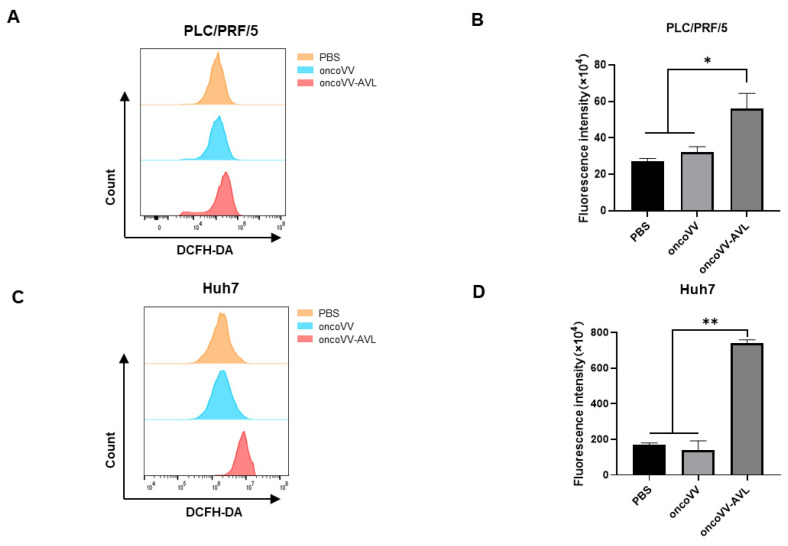
The recombinant virus oncoVV-AVL promoted ROS levels in HCC cells. Analysis of intracellular ROS levels used DCFH-DA staining and flow cytometry. PLC/PRF/5 (**A**,**B**) and Huh7 (**C**,**D**) cells were treated with PBS, oncoVV (MOI = 2), and oncoVV-AVL (MOI = 2), respectively, for 36 h. (**B**,**D**) The bar graphs represent the median fluorescence intensity. (* *p* < 0.05, ** *p* < 0.01).

To determine whether oncoVV-AVL infection induced ROS production in HCC cells, PLC/PRF/5 and Huh7 cells were infected with oncoVV-AVL, at a multiplicity of infection (MOI) of 2. After infection in 36 hours, cells were stained with the fluorogenic marker (DCFH-DA), which is cleaved by intracellular esterases within the cell, producing a fluorescent signal. DCF oxidation was determined by the change in median fluorescence intensity in control infected cells. Compared to the PBS and oncoVV groups, ROS increased more than two times in PLC/PRF/5 cells (Figure 1A,B) and four times in Huh7 cells (Figure 1C,D) by oncoVV-AVL. These results indicated that oncoVV-AVL significantly increased intracellular ROS generation in PLC/PRF/5 and Huh7 cells. 

### 2.2. OncoVV-AVL May Increase ROS Production by Metabolic Reprogramming in HCC Cells

**Figure 2:**
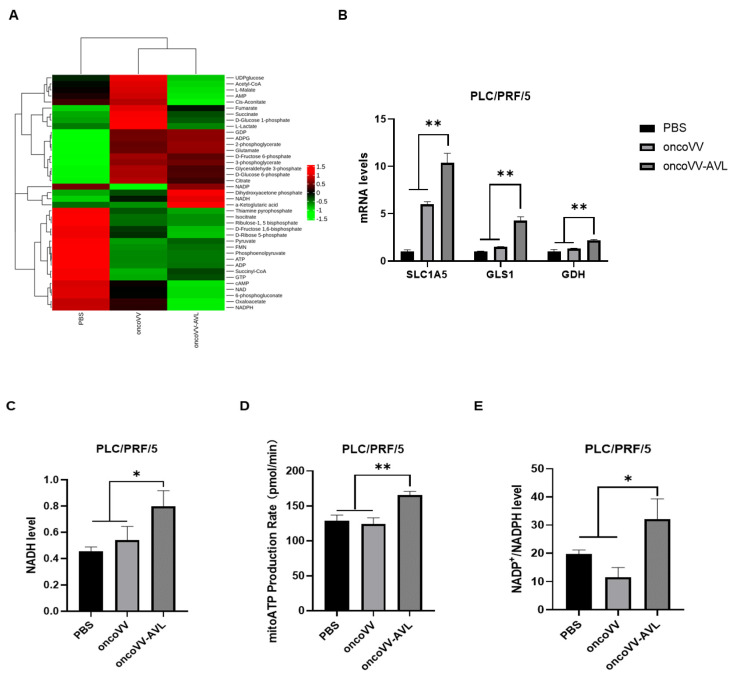
The recombinant virus oncoVV-AVL regulated metabolic reprogramming and promoted the TCA circle. Changes in metabolism in PLC/PRF/5 cells infected with oncoVV-AVL. (**A**) Metabolite heat map of PLC/PRF/5 cells. Before targeted metabolomics analysis, cells were subjected to PBS, oncoVV (MOI = 2), and oncoVV-AVL (MOI = 2) for 18 hours, respectively. (**B**) mRNA levels of target gene SLC1A5, GDH, and GLS1 in PLC/PRF/5 were detected by RT-qPCR. (**C**) Intracellular levels of NADH were measured by the NAD^+^/NADH assay kit with WST-8. WST-8 was used to investigate the effect of oncoVV-AVL on intracellular NADH. After infection in 18 hours at 2 MOI, PLC/PRF/5 was lysed, then the acetaldehyde dehydrogenase working solution and the color development solution were successively added and incubated in the dark. (**D**) The mitoATP was detected by seahorse XF real-time ATP rate assay kit. PLC/PRF/5 cells were infected at 12 h p.i., then added oligomycin and ROT/AA to cell and detected with seahorse XFe 24. (**E**) Intracellular levels of NADP^+^/NADPH were measured using NADP^+^/NADPH assay kit with WST-8. (* *p* < 0.05, ** *p* < 0.01).

ROS is a by-product of oxidative phosphorylation. To confirm that oncoVV-AVL promoted ROS production through reprogramming metabolism, we performed a metabolomic analysis. Cell samples were collected after infection for 18 hours at 2 MOI. As shown in Figure 2A, compared to oncoVV, a significant increase in α-KG, NADH, and ATP was observed in the oncoVV-AVL group, indicating that AVL improved the TCA cycle and oxidative phosphorylation. To explore how AVL increased TCA flux, RT-qPCR detected SLC1A5, GLS1, and GDH mRNA levels. As shown in Figure 2B, the mRNA level of SLC1A5, GLS1, and GDH increased significantly at 6 h post infection (p.i.) compared to oncoVV in PLC/PRF/5. Therefore, oncoVV-AVL may increase the metabolic flux of the TCA cycle by increasing glutamine uptake.

To further confirm changes in NADH and mitoATP levels, we used the NAD^+^/NADH assay kit with WST-8 and seahorse XF real-time ATP rate assay kit, respectively. As shown in Figure 2C–D, the mitoATP and NADH content was increased in oncoVV-AVL-infected PLC/PRF/5 cells. These results indicated that oncoVV-AVL may increase ROS production by enhancing oxidative phosphorylation in HCC cells. 

NADPH provided the reducing power for intracellular antioxidant processes [27], which is an ROS scavenger. PLC/PRF/5 cells were treated with the virus in 18 hours at 2 MOI. The G6PD working solution and the color development solution were added to the lysed cells and incubated away from light. As shown in Figure 2E, the NADP^+^/NADPH level in the oncoVV-AVL-infected cells significantly decreased by approximately three times at 18 h p.i. These results suggested that the elevated NADP^+^/NADPH ratio reduced intracellular reducing power and thus elevated ROS levels.

### 2.3. NADPH Inhibited OncoVV-AVL Replication in HCC Cells

**Figure 3:**
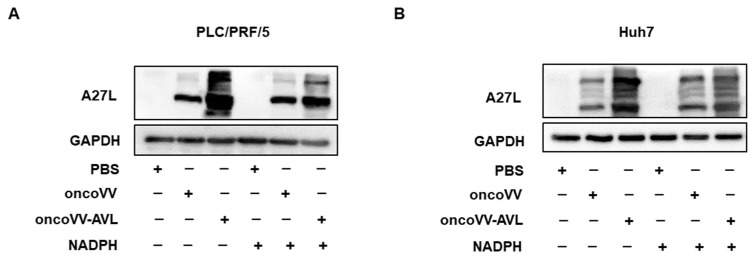
NADPH inhibited the recombinant virus oncoVV-AVL replication. (**A**,**B**) Western blot of A27L was used to address viral replication. PLC/PRF/5 (**A**) and Huh7 (**B**) cells were treated with PBS, oncoVV (MOI = 2), oncoVV-AVL (MOI = 2), or in combination with NADPH (100 uM) for 36 h prior to protein extraction. GAPDH was measured as a loading control.

NAPDH is an important reducing agent in biosynthesis, which plays an important role in ROS elimination [28]. A27L, the mature surface protein of the vaccinia virus, can be used to evaluate viral replication [29]. Western blot was used to investigate the relationship between viral replication and ROS in PLC/PRF/5 and Huh7 cells. As shown in Figure 3A,B, in both PLC/PRF/5 and Huh7 cells, NADPH reduced the expression of A27L in the oncoVV-AVL group but had no effect in the control group. These data indicated that NADPH inhibited oncoVV-AVL replication in HCC cells. 

### 2.4. OncoVV-AVL May Promote Lipid Synthesis by Regulating ROS Levels

**Figure 4:**
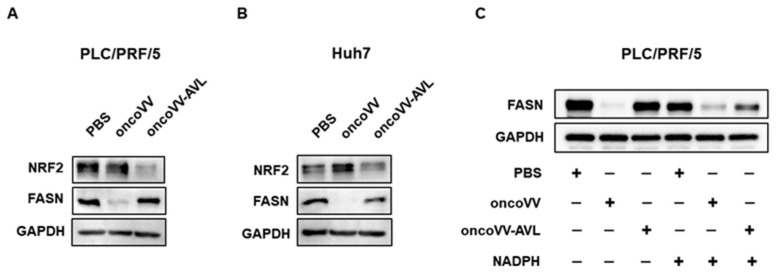
Recombinant virus oncoVV-AVL promoted lipid synthesis by regulating the ROS/NRF2/FASN signaling pathway. The expression levels of NRF2 and FASN in PLC/PRF/5 (**A**,**C**) and Huh7 (**B**) cells were detected by Western blot at 36 h p.i. (**C**) PLC/PRF/5 cells were treated with PBS, oncoVV (MOI = 2), oncoVV-AVL (MOI = 2), or in combination with NADPH (100 uM). GAPDH was measured as a loading control.

The viral envelope is composed of lipids, and sufficient lipids are necessary for the virus to enter the host cell [30]; our previous research found that inhibition of lipid synthesis reduced oncoVV-AVL replication [31]. Fatty acid synthase (FASN) served as a key enzyme parameter associated with the de novo fatty acid synthesis pathway [32]. As shown in Figure 4A–B, in PLC/PRF/5 and Huh7 cells, FASN was significantly up-regulated by oncoVV-AVL compared to the control at 36 h p.i. As shown in Figure 4C, the expression level of FASN protein was down-regulated in PLC/PRF/5 cells after treatment with oncoVV-AVL in combination with NADPH, oncoVV-AVL being the control group. Studies have shown that nuclear factor erythroid 2-related factor 2(NRF2) mediated antioxidant response in the cells and led to a gradual decrease in ROS level [33]. As shown in Figure 4A, in PLC/PRF/5 and Huh7 cells, oncoVV-AVL reduced the expression of NRF2 at 36 h p.i. These results demonstrated that oncoVV-AVL promotes lipid synthesis by regulating the antioxidant network to enhance viral replication.

### 2.5. NADPH Inhibited the Apoptosis of OncoVV-AVL-Induced HCC Cells

**Figure 5:**
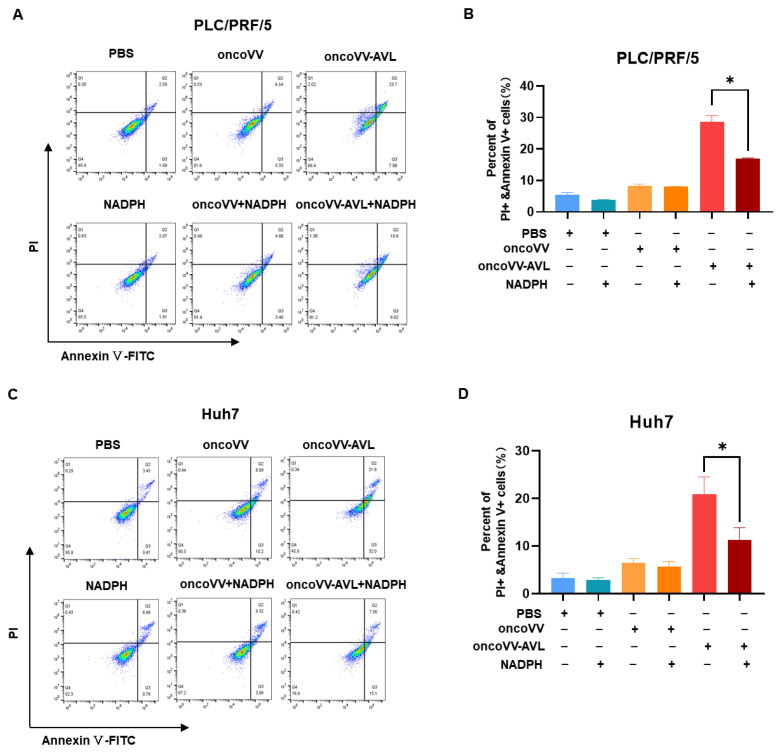
NADPH inhibited the apoptosis of oncoVV-AVL-induced HCC cells. Apoptosis was detected by Annexin/PI staining and flow cytometry. PLC/PRF/5 (**A**,**B**) and Huh7 (**C**,**D**) cells were treated with PBS, oncoVV (MOI = 2), oncoVV-AVL (MOI = 2), or in combination with NADPH (100 uM) at 48 h p.i. (**B**,**D**) Percentage of apoptosis in HCC cells (* *p* < 0.05).

To verify the effect of NADPH on apoptosis of HCC cells, PLC/PRF/5 cells were treated with the virus (MOI = 2) at 48 h p.i., and Huh7 cells were treated with the virus (MOI = 2) at 60 h p.i. The cells were then stained with the fluorogenic marker (Annexin-FITC/PI). Apoptosis was determined by comparing the overall changes in early and late apoptosis changes in infected cells. The percentage of apoptotic cells decreased from 28.69% to 16.93% in PLC/PRF/5 cells (Figure 5A,B) and from 50.67% to 21.96% in Huh7 cells (Figure 5C,D). Our results indicated that NADPH treatment significantly inhibited apoptosis of PLC/PRF/5 and Huh7 cells infected with the recombinant virus oncoVV-AVL.

### 2.6. Molecular Mechanism of OncoVV-AVL-Induced Apoptosis in HCC Cells

**Figure 6:**
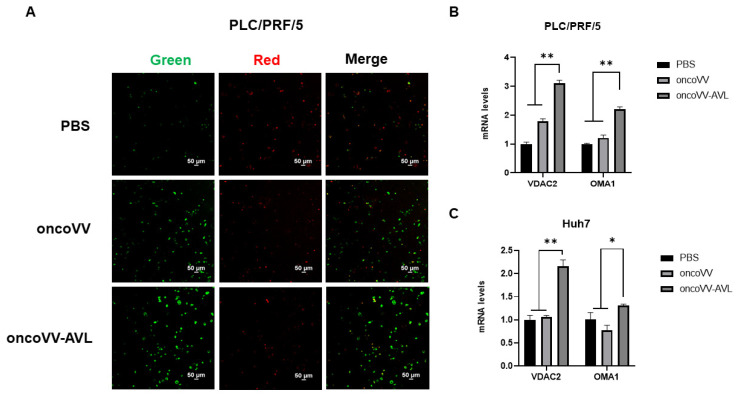
The recombinant virus oncoVV-AVL decreased mitochondrial membrane potential (MMP) in HCC cells and initiated the expression of genes related to apoptosis. (**A**) MMP was measured using JC-1 and confocal laser microscopy. Red fluorescence: JC-1 aggregates; green fluorescence: JC-1 monomer. PLC/PRF/5 cells were treated with PBS, oncoVV (MOI = 2), and oncoVV-AVL (MOI = 2) for 30 h p.i. (**B**,**C**) The mRNA levels of target genes VADC2 and OMA1 in PLC/PRF/5 and Huh7 cells were detected by RT-qPCR at 36 h p.i. (* *p* < 0.05, ** *p* < 0.01).

Research has shown that excessive accumulation of ROS can cause mitochondrial proton leakage, which decreases the mitochondrial membrane potential (MMP) [34]. The decrease in MMP increased mitochondrial membrane permeability. Signals released by mitochondrial outer membrane permeabilization are critical for controlled cell death [35]. To determine the effect of oncoVV-AVL on the MMP of HCC cells, PLC/PRF/5 cells were infected with oncoVV-AVL at an MOI of 2. After infection in 30 h, PLC/PRF/5 cells were loaded with fluorescent probe (JC-1). JC-1 emitted green fluorescence as monomers in the cytoplasm due to reduced membrane potential. As shown in Figure 6A, the MMP of oncoVV-AVL was significantly lower than oncoVV in PLC/PRF/5 cells.

To further investigate the molecular mechanisms underlying apoptosis induced by oncoVV-AVL, RT-qPCR assessed mRNA levels of apoptosis-related factors VADC2 and OMA1. VDAC is a regulator of pores formed by inner mitochondrial membrane proteins, which can assist Bax in binding to mitochondria and forming pores in the mitochondrial membrane, leading to cell death [36,37]. OPA1 is an endogenous dynamin in mitochondria and has an inhibitory effect on apoptosis. OMA1 is activated by mitochondrial membrane depolarization and other cellular stresses, which mediate OPA1 degradation [38]. As shown in Figure 6B, C, in contrast with oncoVV, the transcription levels of VDAC2 and OMA1 in PLC/PRF/5 cells increased significantly at 36 h p.i., which was further confirmed in Huh7 cells. Therefore, our results demonstrated that the membrane potential of HCC cells infected with oncoVV-AVL was depolarized and that the increase in the pore size of the mitochondrial membrane can induce mitochondrial apoptosis.

## 3. Discussion

**Figure 7:**
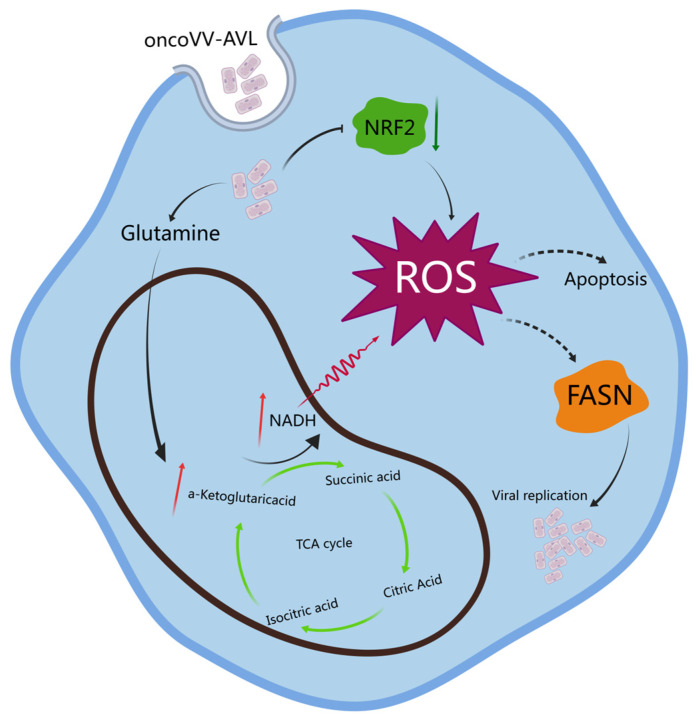
OncoVV-AVL induced ROS to kill HCC cells.

Our previous studies have shown that oncoVV-AVL has remarkable replication and antitumor effects. Specifically, AVL can regulate signaling pathways within tumor cells to enhance the role of oncoVV in killing HCC tumors. As shown in Figure 7, in this study, we found that AVL may mediate the reduction/oxidation (REDOX) system to enhance oncoVV replication and induce apoptosis of HCC cells.

OncoVV-AVL may promote ROS production by remodeling metabolism. ROS originate from the membrane of mitochondria and are the product of mitochondrial respiration [39]. Two mechanisms have been identified as responsible for the increase of ROS: (i) Increasing in the circulating TCA flux. The process enhances the use of NADH to supply electrons to the electron transport chain and promotes the production of ROS and ATP. (ii) Instability of the electron transport chain. That is, when electrons pass through the ETC, a small fraction escapes and prematurely reacts with molecular oxygen, resulting in reduced mitochondrial ATP production. In our investigation, the increases in ATP and NADH was measured, so we hypothesized that oncoVV-AVL enhanced ROS production in the first way. Furthermore, we also found that glutamine transport, glutaminase, and glutamate dehydrogenase were up regulated, suggesting that oncoVV-AVL increased TCA flux through glutamine metabolism. Alongside the mitochondrial enzymes involved in the respiratory chain, NADPH oxidases (NOXs) are recognized as the primary generators of reactive oxygen species (ROS) within cells [40]. NADPH is a substrate for NADPH oxidases (NOXs), which acts as electron donors to activate NOX and thus facilitates the production of ROS [28,41]. We found that inhibition of NADPH oxidase activity had no significant effect on viral replication (Figure S2), whereas addition of substrates with NADPH oxidase activity inhibited oncoVV-AVL replication. This further confirms our inference that after invasion of oncoVV-AVL into hepatocellular carcinoma cells, the production of ROS is dependent on enzymes of the mitochondrial respiratory chain rather than by NOXs. At this point, the main role of NADPH is not to provide raw material for ROS, but to provide reducing power. In addition, oncoVV-AVL also disrupted the intracellular reduction potential by increasing the NADP^+^/NADPH ratio, which created conditions for the presence of ROS.

OncoVV-AVL promoted ROS production to facilitate viral replication. It is reported that the guanylyl transferase activity of alphavirus nsP1 and flavivirus NS5 was enhanced under oxidative conditions, which was conducive to viral replication [42]. SUMOylation of salmon anemia virus proteins in an oxidative environment improved virus output [43]. ROS enhanced EV-A71 replication by decreasing the expression of sirtuin1 and erythroid 2 related nuclear factor [44]. Influenza A virus infection of lung epithelial cells significantly increased ROS by up-regulation of NOX4 and activated MAPK to promote nuclear output of viral ribonucleoproteins and viral release [45]. The viral envelope comprised lipids, and sufficient lipids were essential for virus invasion into host cells [30]. Our previous research found that inhibition of lipid synthesis reduced the ability of oncoVV-AVL replication [31]. FASN is a key enzyme parameter associated with the de novo fatty acid synthesis pathway [32]. This study confirmed that oncoVV-AVL up-regulated ROS to promote lipid synthesis. In summary, oncoVV-AVL may promote viral replication by modulating ROS levels to enhance lipid synthesis. However, the molecular mechanism of ROS that affects lipid synthesis remained to be further explored. 

OncoVV-AVL may induce apoptosis of HCC cells by increasing ROS levels. Our results showed that oncoVV-AVL caused a decrease in mitochondrial membrane potential in HCC cells at a later stage. It is suggested that ROS concentration exceeded the threshold with continuous accumulation of ROS, leading to mitochondrial damage and inducing apoptosis. Excess ROS may lead to the decoupling of the electron transport chain of mitochondria, a decrease in ATP levels, and an increase in the expression of pro-apoptotic protein Bax. Eventually, the outer membrane of mitochondria breaks down, leading to apoptosis. ROS mediated the oligomerization of the pore-forming protein of the mitochondrial membrane pore-forming protein VDAC2 [46], which was beneficial for its help in the formation of pores on the mitochondrial membrane by Bax [37], and activation of OMA1 led to the degradation of the mitochondrial apoptosis inhibitor protein OPA1 [47]. Combined with increased mRNA levels of VDAC2 and OMA1, it was inferred that ROS could mediate the mitochondrial apoptosis pathway by affecting mitochondrial membrane potential and the expression of membrane-associated proteins.

## 4. Materials and Methods

### 4.1. Cells and Viruses 

HEK293A and hepatocellular carcinoma cell lines PLC/PRF/5 and Huh7 were supplied by Hangzhou Qiannuo Biotechnology Co., Ltd. (Hangzhou, China). Cells were cultured in DMEM medium (Gibco, Thermo Fisher Scientific, Waltham, MA, USA) supplemented with 10% fetal bovine serum (FBS, Gibco) and 1% penicillin-streptomycin solution. The control virus oncoVV and the target virus oncoVV-AVL were cultivated in HEK293A and purified with differential centrifugation and stored by our research group. 

### 4.2. Flow Cytometry Analysis for ROS

ROS levels were measured using DCFH-DA (Solarbio, D6470). Cells were cultivated in 6-well plates overnight; thereafter, they were subjected to PBS, oncoVV (MOI = 2), and oncoVV-AVL (MOI = 2) infection for 36 h p.i. DCFH-DA (1:2000 dilution) was utilized to stain the cells suspended in PBS for 20 minutes, then examined through a flow cytometer (AccuriC6, BD Biosciences, San Jose, CA, USA).

### 4.3. Western Blot Analysis

An amount of 2% SDS as the cell lysis buffer was used to resuspend the cell samples, which were then ultrasonicated to disrupt them. The detailed steps of Western blot are referred to the article of our research group [48]. The primary antibody GAPDH(CST,2118S) was purchased from Cell Signaling Technology. A27L (Abcam, ab35219) were purchased from Abcam, NRF2 (Proteintech, 16396-1-AP), and FASN (Proteintech, 10624-2-AP) were purchased from Proteintech. The secondary antibody HRP Goat Anti-Rabbit lgG (H+L) (ABclonal, AS014) were purchased from Abclonal.

### 4.4. Quantitative Real-Time PCR

The cells underwent viral infection for the specified durations. RNA was isolated utilizing the RNA-Quick Purification Kit from ES Science in Shanghai, China, and subsequently converted to cDNA using the Rever Tra Ace qPCR RT Kit from TOYOBO, Japan. This cDNA then underwent amplification with the aid of ChamQ Universal SYBR qPCR Master Mix from Vazyme, based in Nanjing, China. Quantitative analysis of specific gene mRNA levels was conducted, with GAPDH employed as the reference gene for normalization.

### 4.5. Targeted Metabolomics Analysis

PLCPRF/5 cells were seeded in 150 mm plates, infected with oncoVV (MOI = 2), and oncoVV-AVL (MOI = 2), and incubated for 18 h p.i. The cells were washed with pre-cooled PBS at 4 °C and subsequently collected into a pre-cooled centrifuge tube using a cell scraper. The specimens were rapidly frozen in liquid nitrogen for a period of 1 minute, stored at −80 °C and transported on dry ice until metabolomic analysis.

### 4.6. Seahorse XF Real-Time ATP Rate Assay

The mitoATP level of PLCPRF/5 cells was detected using seahorse XF real-time ATP rate assay. PLCPRF/5 cells were seeded in seahorse XF24 culture plate and infected with the virus for 12 h p.i. Oligomycin and ROT/AA were then added to cell and detected with seahorse XFe 24. (Agilent, Santa Clara, CA, USA).

### 4.7. WST-8 Assay

The levels of NADH and NADP^+^/NADPH ratio in PLC/PRF/5 cells were assessed using the WST-8 assay. PLCPRF/5 cells were seeded in 60 mm plates and treated with PBS, oncoVV-AVL at 2 MOI for 18 h p.i. The samples were added to a 96-well plate and subsequently incubated for 10 min with the addition of an acetaldehyde dehydrogenase working solution or G6PD working solution. Then, 10 µL color development solution was introduced into the wells and allowed to incubate for 30 minutes. The absorbance at 450 nm was determined using a microplate reader (Multiskan, Thermo Scientific, Waltham, MA, USA). 

### 4.8. MMP Assay

Cells (5 × 10^5^/well) were seeded in a 15 mm confocal dish and infected with the virus for 30 h p.i. After discarding the medium, the cells were washed with PBS three times and then stained with JC-1 (1:1000 dilution) for 20 min following the manufacturer’s instructions. Finally, cells were subjected to fluorescence microscopy to visualize the potential of the mitochondrial membrane.

### 4.9. Flow Cytometry Analysis for Apoptosis

Apoptotic activity was quantified employing flow cytometry. Following infection by the virus for 48 h p.i. in PLC/PRF/5 cells and 60 h p.i. in Huh7 cells, cells were harvested, resuspended in PBS, and labeled with Annexin V-FITC and propidium iodide (PI) as per the guidelines provided by BD Biosciences (San Jose, CA, USA). The prepared cellular specimens were then subjected to evaluation by flow cytometer (AccuriC6, BD Biosciences, San Jose, CA, USA).

### 4.10. Statistical Analysis

GraphPad Prism 9.0 software was used for the statistical analyses. All data were presented as means ± SEM, and *p* < 0.05 was considered statistically significant. The significance in all figures is indicated as follows: *, *p* < 0.05; **, *p* < 0.01.

## 5. Conclusions

This study has shown that oncoVV-AVL may reprogram the metabolism of HCC cells to promote ROS, and elevated ROS subsequently promoted viral replication and induced apoptosis. This study is conducive to further understanding the replication mechanism and action mechanism of oncoVV-AVL in cancer cells and provides the possibility for the further development and utilization of oncoVV-AVL.

## Data Availability

Data are contained within the article.

## Supplementary Materials

The following supporting information can be downloaded at: https://www.mdpi.com/article/10.3390/md22070307/s1, Figure S1 Gene AVL in PLC/PRF/5 cells was detected by agarose gel electrophoresis. Figure S2. The replication of oncoVV-AVL in combination with a NADPH oxidase inhibitor (Apocynin) in PLC/PRF/5 cells.

## References

[1] Lakhtin V., Lakhtin M., Alyoshkin V. (2011). Lectins of living organisms. The overview. Anaerobe.

[2] Li M., Tian M., Jiang X., Liu Y., Wang Y., Li Y. (2022). Inhibition of galectin-3 ameliorates high-glucose-induced oxidative stress and inflammation in ARPE-19 cells. Cutan. Ocul. Toxicol..

[3] Wang W., Mao S., Yu H., Wu H., Shan X., Zhang X., Cui G., Liu X. (2019). Pinellia pedatisecta lectin exerts a proinflammatory activity correlated with ROS-MAPKs/NF-κB pathways and the NLRP3 inflammasome in RAW264.7 cells accompanied by cell pyroptosis. Int. Immunopharmacol..

[4] Zhang L., Cheng L., Wang Q., Zhou D., Wu Z., Shen L., Zhang L., Zhu J. (2015). Atorvastatin protects cardiomyocytes from oxidative stress by inhibiting LOX-1 expression and cardiomyocyte apoptosis. Acta Biochim. Biophys. Sin..

[5] Liu B., Cheng Y., Zhang B., Bian H.J., Bao J.K. (2009). Polygonatum cyrtonema lectin induces apoptosis and autophagy in human melanoma A375 cells through a mitochondria-mediated ROS-p38-p53 pathway. Cancer Lett..

[6] Müller W.E., Schröder H.C., Skorokhod A., Bünz C., Müller I.M., Grebenjuk V.A. (2001). Contribution of sponge genes to unravel the genome of the hypothetical ancestor of Metazoa (Urmetazoa). Gene.

[7] Gundacker D., Leys S.P., Schröder H.C., Müller I.M., Müller W.E. (2001). Isolation and cloning of a C-type lectin from the hexactinellid sponge Aphrocallistes vastus: A putative aggregation factor. Glycobiology.

[8] Chowaniec H., Ślubowska A., Mroczek M., Borowczyk M., Braszka M., Dworacki G., Dobosz P., Wichtowski M. (2024). New hopes for the breast cancer treatment: Perspectives on the oncolytic virus therapy. Front. Immunol..

[9] Huang Z., Guo H., Lin L., Li S., Yang Y., Han Y., Huang W., Yang J. (2023). Application of oncolytic virus in tumor therapy. J. Med. Virol..

[10] Manocha E., Caruso A., Caccuri F. (2021). Viral Proteins as Emerging Cancer Therapeutics. Cancers.

[11] Chi P.I., Huang W.R., Chiu H.C., Li J.Y., Nielsen B.L., Liu H.J. (2018). Avian reovirus σA-modulated suppression of lactate dehydrogenase and upregulation of glutaminolysis and the mTOC1/eIF4E/HIF-1α pathway to enhance glycolysis and the TCA cycle for virus replication. Cell. Microbiol..

[12] Carinhas N., Koshkin A., Pais D.A., Alves P.M., Teixeira A.P. (2017). (13) C-metabolic flux analysis of human adenovirus infection: Implications for viral vector production. Biotechnol. Bioeng..

[13] Yu Y., Clippinger A.J., Alwine J.C. (2011). Viral effects on metabolism: Changes in glucose and glutamine utilization during human cytomegalovirus infection. Trends Microbiol..

[14] Greseth M.D., Traktman P. (2014). De novo fatty acid biosynthesis contributes significantly to establishment of a bioenergetically favorable environment for vaccinia virus infection. PLoS Pathog..

[15] Purandare N., Ghosalkar E., Grossman L.I., Aras S. (2023). Mitochondrial Oxidative Phosphorylation in Viral Infections. Viruses.

[16] Foo J., Bellot G., Pervaiz S., Alonso S. (2022). Mitochondria-mediated oxidative stress during viral infection. Trends Microbiol..

[17] Combs J.A., Norton E.B., Saifudeen Z.R., Bentrup K.H.Z., Katakam P.V., Morris C.A., Myers L., Kaur A., Sullivan D.E., Zwezdaryk K.J. (2020). Human Cytomegalovirus Alters Host Cell Mitochondrial Function during Acute Infection. J. Virol..

[18] Claus C., Schönefeld K., Hübner D., Chey S., Reibetanz U., Liebert U.G. (2013). Activity increase in respiratory chain complexes by rubella virus with marginal induction of oxidative stress. J. Virol..

[19] Li M., Zhang M., Ye Q., Liu Y., Qian W. (2023). Preclinical and clinical trials of oncolytic vaccinia virus in cancer immunotherapy: A comprehensive review. Cancer Biol. Med..

[20] Qian M., Tan H.M., Yu N., Wang T., Zhang Q. (2018). Inactivated Sendai Virus Induces ROS-dependent Apoptosis and Autophagy in Human Prostate Cancer Cells. Biomed. Environ. Sci. BES.

[21] Lin P.Y., Liu H.J., Chang C.D., Chang C.I., Hsu J.L., Liao M.H., Lee J.W., Shih W.L. (2011). Avian reovirus S1133-induced DNA damage signaling and subsequent apoptosis in cultured cells and in chickens. Arch. Virol..

[22] Deng L., Chen M., Tanaka M., Ku Y., Itoh T., Shoji I., Hotta H. (2015). HCV upregulates Bim through the ROS/JNK signalling pathway, leading to Bax-mediated apoptosis. J. Gen. Virol..

[23] Burton E.M., Voyer J., Gewurz B.E. (2022). Epstein-Barr virus latency programs dynamically sensitize B cells to ferroptosis. Proc. Natl. Acad. Sci. USA.

[24] Yang X., Liu X., Nie Y., Zhan F., Zhu B. (2023). Oxidative stress and ROS-mediated cellular events in RSV infection: Potential protective roles of antioxidants. Virol. J..

[25] Wu T., Xiang Y., Liu T., Wang X., Ren X., Ye T., Li G. (2019). Oncolytic Vaccinia Virus Expressing Aphrocallistes vastus Lectin as a Cancer Therapeutic Agent. Mar. Drugs.

[26] Jiang R., Zhang X., Zhou N., Jia X., Chen K., Zhou Y., Ye T., Li G. (2022). Oncolytic Vaccinia Virus Harboring Aphrocallistes vastus Lectin Inhibits the Growth of Hepatocellular Carcinoma Cells. Mar. Drugs.

[27] Xiao W., Loscalzo J. (2020). Metabolic Responses to Reductive Stress. Antioxid. Redox Signal..

[28] Zhang Y.L., An Y., Sun L.J., Qu H.L., Li X., He X.T., Wu R.X., Chen F.M., Tian B.M., Yin Y. (2023). NADPH-dependent ROS accumulation contributes to the impaired osteogenic differentiation of periodontal ligament stem cells under high glucose conditions. Front. Endocrinol..

[29] Sanderson C.M., Hollinshead M., Smith G.L. (2000). The vaccinia virus A27L protein is needed for the microtubule-dependent transport of intracellular mature virus particles. J. Gen. Virol..

[30] Allen C.N.S., Arjona S.P., Santerre M., Sawaya B.E. (2022). Hallmarks of Metabolic Reprogramming and Their Role in Viral Pathogenesis. Viruses.

[31] Zhou Y., Wang Q., Ying Q., Zhang X., Chen K., Ye T., Li G. (2023). Effects of Oncolytic Vaccinia Viruses Harboring Different Marine Lectins on Hepatocellular Carcinoma Cells. Int. J. Mol. Sci..

[32] Zhang J., Song Y., Shi Q., Fu L. (2021). Research progress on FASN and MGLL in the regulation of abnormal lipid metabolism and the relationship between tumor invasion and metastasis. Front. Med..

[33] Ferrari M., Zevini A., Palermo E., Muscolini M., Alexandridi M., Etna M.P., Coccia E.M., Fernandez-Sesma A., Coyne C., Zhang D.D. (2020). Dengue Virus Targets Nrf2 for NS2B3-Mediated Degradation Leading to Enhanced Oxidative Stress and Viral Replication. J. Virol..

[34] Brookes P.S. (2005). Mitochondrial H(+) leak and ROS generation: An odd couple. Free Radic. Biol. Med..

[35] Jeong S.Y., Seol D.W. (2008). The role of mitochondria in apoptosis. BMB Rep..

[36] Naghdi S., Hajnóczky G. (2016). VDAC2-specific cellular functions and the underlying structure. Biochim. Biophys. Acta.

[37] Chin H.S., Li M.X., Tan I.K.L., Ninnis R.L., Reljic B., Scicluna K., Dagley L.F., Sandow J.J., Kelly G.L., Samson A.L. (2018). VDAC2 enables BAX to mediate apoptosis and limit tumor development. Nat. Commun..

[38] Wu Z., Zuo M., Zeng L., Cui K., Liu B., Yan C., Chen L., Dong J., Shangguan F., Hu W. (2021). OMA1 reprograms metabolism under hypoxia to promote colorectal cancer development. EMBO Rep..

[39] Lennicke C., Cochemé H.M. (2021). Redox metabolism: ROS as specific molecular regulators of cell signaling and function. Mol. Cell.

[40] Bedard K., Krause K.H. (2007). The NOX family of ROS-generating NADPH oxidases: Physiology and pathophysiology. Physiol. Rev..

[41] de Jesus D.S., Bargi-Souza P., Cruzat V., Yechoor V., Carpinelli A.R., Peliciari-Garcia R.A. (2022). BMAL1 modulates ROS generation and insulin secretion in pancreatic β-cells: An effect possibly mediated via NOX2. Mol. Cell. Endocrinol..

[42] Gullberg R.C., Jordan Steel J., Moon S.L., Soltani E., Geiss B.J. (2015). Oxidative stress influences positive strand RNA virus genome synthesis and capping. Virology.

[43] Fredericksen F., Villalba M., Maldonado N., Payne G., Torres F., Olavarría V.H. (2019). Sumoylation of nucleoprotein (NP) mediated by activation of NADPH oxidase complex is a consequence of oxidative cellular stress during infection by Infectious salmon anemia (ISA) virus necessary to viral progeny. Virology.

[44] Li H., Bai Z., Li C., Sheng C., Zhao X. (2020). EV71 infection induces cell apoptosis through ROS generation and SIRT1 activation. J. Cell. Biochem..

[45] Amatore D., Sgarbanti R., Aquilano K., Baldelli S., Limongi D., Civitelli L., Nencioni L., Garaci E., Ciriolo M.R., Palamara A.T. (2015). Influenza virus replication in lung epithelial cells depends on redox-sensitive pathways activated by NOX4-derived ROS. Cell. Microbiol..

[46] Zhu Z., Zhou X., Du H., Cloer E.W., Zhang J., Mei L., Wang Y., Tan X., Hepperla A.J., Simon J.M. (2023). STING Suppresses Mitochondrial VDAC2 to Govern RCC Growth Independent of Innate Immunity. Adv. Sci..

[47] Noh S., Phorl S., Naskar R., Oeum K., Seo Y., Kim E., Kweon H.S., Lee J.Y. (2020). p32/C1QBP regulates OMA1-dependent proteolytic processing of OPA1 to maintain mitochondrial connectivity related to mitochondrial dysfunction and apoptosis. Sci. Rep..

[48] Ni J., Feng H., Xu X., Liu T., Ye T., Chen K., Li G. (2021). Oncolytic Vaccinia Virus Harboring Aphrocallistes vastus Lectin Inhibits the Growth of Cervical Cancer Cells Hela S3. Mar. Drugs.

