# Supplementary material for: ROS Induced by Aphrocallistes vastus Lectin Enhance Oncolytic Vaccinia Virus Replication and Induce Apoptosis in Hepatocellular Carcinoma Cells
Source: Mar Drugs. 2024 Jun 30;22(7):307. doi: 10.3390/md22070307 (PMC11278381; doi:10.3390/md22070307)
Supplement: Supplementary file 1 [file marinedrugs-22-00307-s001.zip › marinedrugs-3016695-supplementary.pdf]

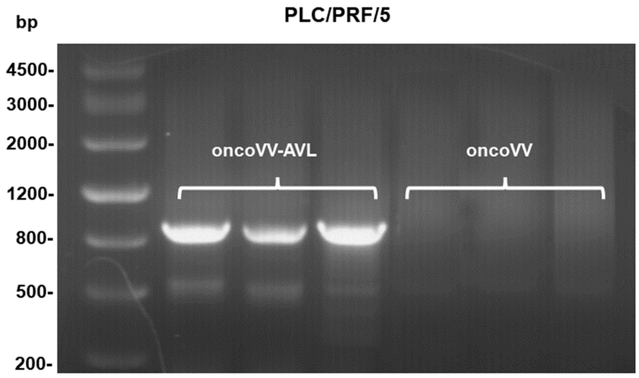

Figure S1. Gene *AVL* in PLC/PRF/5 cells was detected by agarose gel electrophoresis.

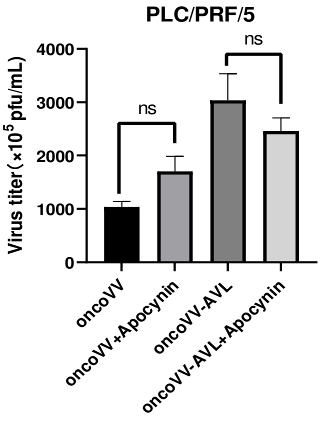

Figure S2. The replication of oncoVV-AVL in combination with a NADPH oxidase inhibitor (Apocynin) in PLC/PRF/5 cells.
